# Polish Version of the Neighbourhood Environment Walkability Scale (NEWS-Poland)

**DOI:** 10.3390/ijerph13111090

**Published:** 2016-11-04

**Authors:** Michał Jaśkiewicz, Tomasz Besta

**Affiliations:** Institute of Psychology, University of Gdańsk, Gdańsk 80-309, Poland; t.besta@ug.edu.pl

**Keywords:** walkability, built environment, assessment, wellbeing, Poland

## Abstract

The characteristics of built environments are the subject of intense consideration in the search for solutions to promote wellbeing and a higher quality of life among the inhabitants of cities. Walkability, defined as the extent to which the built environment is friendly to living and fulfilling the needs of the area, has become an important concept in sustainable urban design, public health and environmental psychology. This study systematically adapted the Neighbourhood Environment Walkability Scale (NEWS) for Poland, and evaluated the construct validity aspects of the adapted version among Polish adults. A total sample of 783 participants from a TriCity (Trójmiasto) agglomeration completed the adapted version of the NEWS. Smaller extracted samples of the participants also completed wellbeing related scales, including self-efficacy, local identity and distance to city centre measures. It was expected that various districts of Gdańsk would differ in terms of walkability. The confirmatory factor analysis showed satisfactory goodness-of-fit statistics and factor loadings corresponding to the proposed original factor structure. According to the predictions, the NEWS subscales correlated with the self-efficacy, local identity and wellbeing related measures. In addition, the comparisons between the neighbourhoods of Gdańsk also showed a predictable pattern of results. Overall, the NEWS demonstrated satisfactory measurement properties, and may be useful in the evaluation of the built environment in Poland.

## 1. Introduction

The characteristics of city architecture and the built environment are related to various aspects of residents’ psychological functioning and wellbeing [1,2]. Several factors, such as the presence of open space and green areas, in a city’s districts are related to the residents’ satisfaction and their health [3,4]. One important factor that has been shown to be associated with better social functioning and physical health is walkability. Several previous studies have revealed the presence of a relationship between the built environment and a willingness to engage in physical activities [5,6]. A more walkable built environment has also been related to a higher perceived quality of life in the city [7].

Walkability, as well as various aspects of the built environment, has also been considered to be related to non-communicable diseases (NCDs), like obesity and diabetes, which are some of the growing health challenges across high and middle-income countries. Moreover, a lack of physical activity and the extensive daily use of a private car could be considered important risk factors in the development of obesity, cardiovascular disease and other NCDs [8]. These diseases are also on the rise in Poland; for example, reports on the development of diabetes have shown that the increase in the number of diabetics in Poland has been greater than that previously forecasted. For example, in 2011, over 1.6 million diabetic patients got medical treatment. Moreover, it has been estimated that, as for prevalence of diabetes, in 2011 over three million people in Poland suffered from its various forms (around one million undiagnosed), and over 21 thousand die each year as a result of diabetes and diabetes-related complications [9]. For interventions aimed at lowering the risk of NCDs, modifications of the built environment in order to encourage more physical activity could be seen as an important pathway [10,11].

To develop more research on the roles of walkable city and town areas in both the physical and psychological health of residents, valid measures of walkability are needed. In the current study, we focused on the presentation of Polish research on the adaptation of the Neighbourhood Environment Walkability Scale (NEWS).

The Neighbourhood Environment Walkability Scale (NEWS) is instrument designed to measure residents’ perceptions of the environmental attributes of their local area [12]. In such context, walkability is defined in terms of the presence in the built environment characteristics that are associated to level of walking among residents. Those characteristics create the local environment as a friendly to living (e.g., aesthetic, safety from traffic) and fulfilling the needs, where the residents may perform their daily activities without using the car.

This scale has been used to measure the walkability in various environments, including by researchers cooperating with the International Physical Activity and the Environment Network in 12 countries [13], and has been adapted to different cultural contexts. Recently, this scale was translated and implemented in countries such as China, Nigeria, and India [14,15,16].

The NEWS is a self-reporting measure based on the participants’ answers to questions related to various aspects of their surrounding environment. The subscales of the NEWS were designed to assess the perceived residential density, land use mix, street connectivity, walking and biking infrastructure, aesthetics, traffic hazards and safety. Previous studies have shown that the NEWS measure was a reliable tool for assessing walkability, despite some country-specific modifications [13].

### 1.1. Overview of the Present Study

The aim of this study was to adapt and validate the NEWS in Polish context. The validation procedures included the construct validity from: (1) the confirmatory factor analysis; (2) the analysis of the correlation between the NEWS, self-efficacy, local identity, affective reactions and perceived quality of life; and (3) the comparisons between various types of neighbourhoods in Gdańsk, Poland. In addition, we explored the relationships between walkability and various measures of psychological functioning. Specifically, we tested whether walkability, as a characteristic of one’s neighbourhood, is related to self-efficacy, local identity, emotions and the quality of life.

### 1.2. Walkability Self-Efficacy

From the perspective of Bandura’s theory, human functioning is considered to be the product of a dynamic interplay between personal, behavioural and environmental influences. Self-efficacy is a person’s belief in his or her ability to succeed in a particular situation. In attempting to investigate how personal and neighbourhood factors work together, researchers have found significant effects for both variables: neighbourhood walkability and self-efficacy on physical activity [17]. Self-efficacy has also been shown to moderate the relationship between access to recreational facilities and physical activity [18].

Bandura’s socio-cognitive theory posits that external environmental conditions do not affect behaviour directly, but through personal perception [19]. The perceived characteristics of the environment may alter cognitions about the ability to fulfil the personal needs in the neighbourhood, but this relationship also depends on individual experiences and personal factors. People are not just products of their life circumstances, they are able to create and reinterpret their environments to maximize the pursuit of their goals [20]. Neighbourhood walkability can be understood as the extent to which the characteristics of the built environment may facilitate residents to access services or recreation. Despite their individual differences in self-efficacy, we assumed that the perception of the neighbourhood as less walkable would be related to lower self-efficacy in the ability to effectively move and fulfil an individual’s daily needs.

### 1.3. Local Identity

The relationship between neighbourhood walkability and identity-related variables has been tested in the context of the quality of life [7]. Neighbourhood walkability has also been linked to social cohesion [21], and a pedestrian-oriented neighbourhood is expected to encourage the residents to interact [22]. Some evidence also suggests that walkable neighbourhood may indirectly influence quality of life through stronger communal identity [7]. The levels of social capital defined as features of social organizations (e.g., trust, and norms) that facilitate coordinated actions are higher in more walkable neighbourhoods, where individuals have better opportunities to interact [22].

Some evidence related to commuting habits also seems to be in accordance with previous findings; for example, commuting more than 30 min correlated to lower levels of social satisfaction in Vienna [23], and individuals commuting more than 60 min in southern Sweden also reported lower levels of social participation [24]. Moreover, political participation has been shown to be higher in communities with walking, biking and public transit [25]. When taking previous findings into consideration, we predicted a positive relationship between neighbourhood walkability and a sense of local identity.

### 1.4. Emotions and Quality of Life

Numerous studies in environmental psychology have suggested a relationship between a neighbourhood’s characteristics and emotions, wellbeing and quality of life, and recent research has shown a link between walkability, the arrangement of space and a sense of the quality of life [22,26,27]. In addition, easy access to convenient public transportation and to cultural and leisure amenities also promotes happiness [28]. Previous research has found that a lower perceived level of neighbourhood crime, indicative of greater neighbourhood walkability, was related to lower symptoms of depression among older Latinos [29]. In a similar vein, the perceived far proximity to facilities via walking [30] and unsafe traffic [31] contributed to depressive symptoms among elders.

Characteristics related to walkability, such as the presence of trees, landscaping views or the proximity to a park, may provide psychological benefits, including stress reduction [32,33], a reduction in the feelings of anger, frustration and aggression, and trigger positive emotions [33]. Moreover, the presence of trees has been shown to have a positive effect on the sense of safety in a neighbourhood [34,35]. Employees with a view of nature from their office windows have reported higher life satisfaction and overall health [36], while a relationship between green exposure and wellbeing has also been demonstrated [37]. Furthermore, the restorative effects of contact with nature have been demonstrated in numerous research studies [38,39,40].

Based on the abovementioned information, we tested the construct validity of the NEWS-Poland scale, and predicted relationships between certain aspects of walkability (especially aesthetics, safety from traffic and safety from crime) with the overall quality of life and pleasant affect experienced in one’s neighbourhood.

### 1.5. Walkability and Neighbourhood Specificity: The Case of Gdańsk

With regard to the aspects of communication and geography, the city of Gdansk is informally divided into Lower and Upper Terraces. These terms are related to the specific physiography divided along the edge of a moraine plateau [38]. Because of this barrier, the earliest spatial development was restricted to the Lower Terrace, whereas the Upper Terrace has been an area of urban and rapid suburban expansion in recent decades. The Lower Terrace refers to those residential districts located in areas with a low altitude, closer to the seashore, along the railway transportation system in the TriCity area (SKM: Fast Urban Railway). The role of the SKM as a link is considered to be a neighbourhood advantage [41]. The residential buildings of the Lower Terrace consist mainly of a large block of flats that was built in the sixties and seventies (20th century) to meet the housing needs related to the rapid urbanization processes. The districts of the Lower Terrace are well-connected by the public transportation system, with better access to the recreational area along the Baltic Seashore. The residential density of the Lower Terrace districts is also higher.

We decided also to analyse separately two historic districts located on the lower terrace: Oliwa (Oliva) and Śródmieście (Downtown). Śródmieście is a historic centre of Gdansk, which is where most of the monuments (e.g., Concatedra St. Mary’s—the Europe’s largest temple built of brick) and public administration buildings are located. The second historical, district Oliwa (Oliva), is considered one of the most charming districts of Gdansk. Oliwa is known for cathedral, oliwa forest complex on the edge of the TriCity Landscape Park, and the most eagerly visited urban park in Gdansk.

The Upper Terrace districts are located on a moraine plateau, and in a suburban area along a bypass of Gdańsk. The residential buildings of the Upper Terrace consist mainly of 4–5 coordinated blocks of flats that were built in recent decades. There is a difference in the price of housing between the Upper and Lower Terraces of Gdansk, and this is one of the main reasons why the cheaper southern districts of the Upper Terrace attracts many people [42]. Similar to many other cities, living in the suburban areas of Gdańsk is associated with traffic jams and difficulties in commuting from there to the inner city [41].

**Figure 1:**
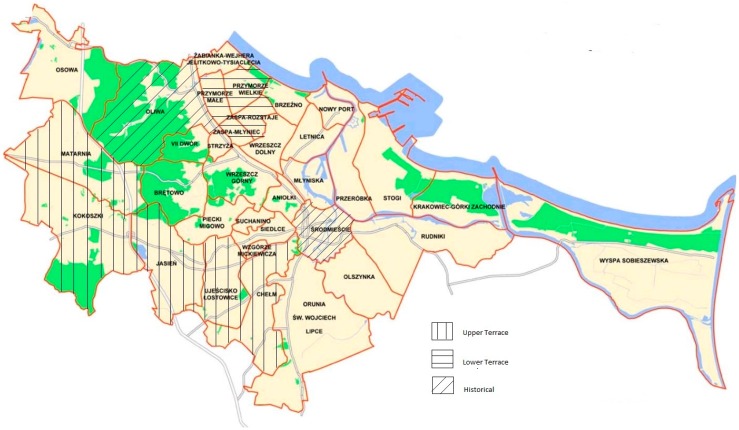
Map of the studied neighbourhoods in Gdańsk.

In order to assess the construct validity of the NEWS-Poland, we decided to make comparisons between the districts of the Lower and Upper Terraces and the historical ones (see Figure 1). We hypothesized that: (1) Lower Terrace areas will be reported as more walkable comparing to more suburban Upper Terrace, and (2) historical districts will be perceived as more aesthetic than areas of Lower and Upper Terrace.

## 2. Materials and Methods

### 2.1. Participants and Procedure

In total, a sample of 783 participants (532 women) who are citizens of the TriCity area (Gdansk, Sopot, Gdynia, Rumia, Reda and Wejherowo) participated in this study. The mean age of the participants was 24.76 years old (SD = 7.89), with a range between 15 and 75 years old. The recruitment was carried out via Internet neighbourhood forums and websites, where the link to the study was placed. The participants included undergraduate students from the University of Gdansk and other adult inhabitants of the urban areas recruited by them. A confirmatory factor analysis was conducted on the total sample (N = 783). Although citizens of the whole TriCity agglomeration were represented in the study, most of them were recruited from the biggest city of Gdańsk, where comparisons between various neighbourhoods was conducted. In the case of other towns, the number of respondents living in the various districts did not allow making such analysis.

To assess the construct validity, we extracted two smaller groups of participants from the total sample, who were asked to fill out additional questionnaires: a single-item quality of life scale, self-efficacy and distance to the city centre (sample 1A), neighbourhood-related emotions and local identity (sample 1B). Sample 1A consisted of 156 participants (111 women), with a mean age of 26.57 years old (SD = 9.38), and sample 1B consisted of 196 participants (120 women), with a mean age of 24.62 years old (SD = 7.13).

### 2.2. Questionnaires

All of the participants were asked to fill out the Polish version of the NEWS-A scale [12]. The adapted NEWS survey consisted of 53 items that assessed the following perceived environmental characteristics: residential density (6 items), distance to facilities (23 items), ease of access to non-residential uses (land use mix access) (3 items), street connectivity (3 items), infrastructure and safety for walking and cycling (6 items), aesthetics (4 items), traffic safety (3 items), safety from crime (3 items) and two single items (cul-de-sacs and physical barriers). In the subscale distance to facilities, the participants were asked to estimate the walking distance to amenities, in which a 5 min walk was coded as 5 and a 30+ min walk was coded as 1.

We used the same 19 items as in the original scale: convenience/small grocery shop, supermarket, hardware store, fruit/vegetable market, clothing store, post office, library, elementary school, other school, book store, fast food restaurant, coffee place, bank, non-fast food restaurant, pharmacy/drug store, salon/barber shop, job or school, park, recreational centre and gym or fitness facility. The following changes were implemented:

- The bus or train stop item was modified for the Gdańsk context as a “bus or tram stop”, and an additional item was created for the SKM or Pomeranian Metropolitan Railway (PKM) train stops.
- The park item was modified to park or forest.
- The laundry/dry cleaning service item was omitted, because in the Polish context these services are usually situated inside shopping malls, and are not commonly used on a weekly basis.
- The video store item was omitted because it is not popular in Poland.
- A beach item was added.

Additionally, the participants in sample 1A were asked to answer:

- A quality of life single-item scale, in which the general quality of life was measured by the question: “How would you rate your quality of life?” The 9-point response scale ranged from 1—very poor to 9—very good [43].
- A walkability self-efficacy scale, in which the self-efficacy was conceptualized as judgments about one’s ability to cope, and the controllability was conceptualized as judgments about one’s personal influence in the urban context. We designed this scale following the guidelines of Bandura [44], which consisted of 4 items (Cronbach’s α = 0.61), for example, “I am able to avoid obstacles (standing too long at traffic lights, tunnels, lack of pavement, excessive traffic and uncomfortable sidewalks), and move quickly and pleasantly within my surroundings” and “I know my neighbourhood well enough that I am able to quickly advise someone else where to go to buy something or to get things done”. The 3 items version of this instrument (Cronbach’s α = 0.0.71) was tested in prior experimental study. As we expected, one-way ANOVA revealed significant differences between groups exposed to photography of walkable and non-walkable areas, *F* (1,71) = 6.25, *p* < 0.001. Participants exposed to car-oriented streets rated their walkability self-efficacy as lower (*M* = 14.53), comparing to participants exposed to pedestrian friendly zones (*M* = 16.33).
- A perceived distance to the city centre single-item scale, in which the participants were asked to indicate on a 7-point scale how far from the city centre they lived. Scale was anchored by 1—very far away from the city centre and 7—very near the centre of the city.

In sample 1B, the participants completed the following measures:

- An affective reaction single-item scale, in which the participants were asked to indicate what kinds of emotions they usually experience in their neighbourhood. They answered on a 9-point scale, with 1—very unpleasant and 9—very pleasant. One-item measures anchored unpleasant–pleasant was previously used in environmental studies to assess affective appraisal of residential area [45] and to investigate the preferences toward urban and natural environment [46].
- The Local Identity Scale [47], in which the participants evaluated how important, from the perspective of who they were and who they felt they were, they found things like their neighbourhood, city and region. They answered on a 5-point scale, with 1—not important at all and 5—extremely important. In this study, Cronbach’s α of the scale was = 0.81.

## 3. Results

### 3.1. Confirmatory Factor Analysis (CFA)

We decided to choose the same common items of the NEWS-A, which have been previously tested in models across 12 countries; therefore, the measurement models, including the following factors and single items, were estimated: land use mix (three items), street connectivity (two items), infrastructure and safety for walking and cycling (six items), aesthetics (four items), traffic safety (three items), safety from crime (three items), not many cul-de-sacs (one item) and physical barriers to walking (one item).

These eight factors were assumed to be inter-correlated.

A confirmatory factor analysis (AMOS) was applied to the sample, to test the hypothesis proposing that the latent variables would account for the relationships between the 25 observed variables. We treated CFI values ≥0.90 as indicative of acceptable levels of model fit, if the other indices met the criteria [10]. The a priori model, based on the item groupings proposed by the developers of the original NEWS-A, adequately fit the observed data (CFI = 0.906, RMSEA = 0.056, 95% CI = 0.051 to 0.060, SRMR = 0.04, χ^2^ = 706.60, df = 205, *p* < 0.001). All of the items also showed higher than desirable standard loadings (>0.30), and the factor loadings are presented in Table 1.

### 3.2. Correlations Analysis

To test the construct validity, we performed an analysis of correlations (Table 2 and Table 3). The results showed that all of the subscales, except the residential density, were significantly related to the walkability self-efficacy (Pearson’s *r* between 0.34 and 0.48) in a predicted direction. The more walkable the neighbourhood, the more the residents felt able to move efficiently and to fulfil their daily needs.

The distance to the facilities, street connectivity, aesthetics and safety from crime were all slightly related to the local identity (Pearson’s *r* between 0.15 and 0.26), and partially confirmed the notion that walkable neighbourhoods are more likely to be perceived as a part of one’s identity.

Three subscales of the NEWS (aesthetics, safety from traffic and safety from crime) were related to both measures of wellbeing: the overall quality of life and experiencing pleasant affect in the neighbourhood (Pearson’s *r* between 0.20 and 0.35), while two other subscales (street connectivity and places for walking and cycling) correlated slightly positively with only the overall quality of life (respectively, *r* = 0.20 and 0.19).

Four of the subscales of neighbourhood walkability (land use mix access, distance to facilities, street connectivity and places for walking and cycling) were related to the perceived distance to the city centre in a predicted direction. Those participants who perceived their neighbourhood as being closer to the city centre rated it as more walkable, with facilities and services at closer distances, with more pedestrian and cyclist friendly streets and smaller architecture.

To test the construct validity, we also performed comparisons between the different neighbourhoods in Gdańsk. We chose two neighbourhoods from the Lower Terrace (Przymorze—LT1 and Zaspa—LT2), two neighbourhoods from the Upper Terrace (Jasień, Matarnia, Kokoszki—UT2 and Chełm, Ujeścisko—UT1) and two historical ones (Sródmieście Downtown—H1, and Oliwa—H2). We conducted a separate one-way ANOVA analysis, with the subscales of the NEWS as a dependent variable. Analysis of variance showed significant differences among neighbourhoods with regard to all of the subscales with exception of safety from crime (see Table 4). To find out which means are significantly different, we conducted the analysis of planned contrasts (comparisons). We decided to make the comparisons between Lower and Upper Terrace neighbourhoods. According to predictions, areas of the Upper Terrace were reported to be significantly less walkable when compared to Lower Terrace with regard to residential density (*t* = 12.01, *p* < 0.001). This pattern of result seemed to be close to the objective Gdańsk City council data on population density [48], as well as to the data on the dominant types of residence [49]. Upper Terrace were also reported to be less walkable with regard to distance to facilities (*t* = 8.00, *p* < 0.001), land use mix access (*t* = 3.33, *p* = 0.001), street connectivity (*t* = 4.34, *p* < 0.001), places for walking and cycling (*t* = 4.96, *p* < 0.001) and aesthetic (*t* = 3.41, *p* = 0.001). We found no significant differences in case of safety from traffic, and safety from crime. Additionally, to assess the construct validity of aesthetic subscale, we compared historical districts (Oliwa and Śródmieście) to Lower and Upper Terrace. As we expected, planned comparisons also revealed that historical districts were rated as significantly more aesthetic than Lower (*t* = 4.05, *p* = 0.001) and Upper Terrace areas (*t* = 6.61, *p* < 0.001).

## 4. Discussion

In this research, we evaluated the reliability of the NEWS questionnaire adapted for Poland, which was revealed to be a reliable tool for accessing walkability in the context of Polish cities. The results from the AMOS analyses provided satisfactory goodness-of-fit statistics and reliability coefficients for a six-factor and two single items model of the NEWS, corresponding to the proposed original factor structure. According to our predictions, the NEWS scores were related to the wellbeing measures of positive effects experienced in one’s neighbourhood, and to the overall quality of life. The three aspects of the neighbourhood walkability that seemed to be especially relevant to an individual’s wellbeing were aesthetics, safety from traffic and safety from crime.

The aesthetic subscale of the NEWS consisted of items related to the presence of trees and natural landscapes; therefore, the results presented may also be interpreted in terms of the green impact on daily functioning. Recently, it was determined that the study participants who used the vegetated pedestrian trail at least once per week had lower hair cortisol levels and were more satisfied with their lives [50]. Because natural environments permit effortless attention and restoration [51], the presence of greenery has been associated with lower crime rates [35], and the presence of natural elements increases an individual’s preference for urban settings [52,53]. Therefore, it is not surprising that in our study the aesthetic aspects of the NEWS were proven to be related to experiencing the pleasant affect, and to the subjective overall quality of life. We also found significant differences between the Gdansk’s neighbourhoods of Śródmieście (Downtown), and Oliwa, with historical buildings like the metropolitan cathedral and being situated near the park and green areas, scored significantly higher on the aesthetics subscale.

Traffic may also affect the wellbeing of a town’s citizens in many ways, including unintentional injuries, hassles of driving and parking, exposure to traffic and to traffic-related noise, vibration and air pollution and a reduction in street activities [54,55]. Those persons reporting traffic stress had lower health statuses [54], and the chronic noise of everyday local traffic has been modestly related to the level of physiological stress [56]. In our study, the safety from traffic subscale was correlated with both the overall quality of life, and experiencing pleasant affect in the neighbourhood. Therefore, the creation of zones free from traffic seems to be an important element of public policy influencing how people feel in their neighbourhood.

With regard to safety, many studies have shown the impact of crime on residential satisfaction and the quality of life. There is also some evidence for a link between contextual factors and post-traumatic stress disorder (PTSD) [31], with surveys indicating that neighbourhood crime and unsafe traffic are associated with depressive symptoms [57], but lower perceived neighbourhood crime served as a protective factor [29]. Despite numerous studies indicating the impact of neighbourhood disorder and fear of crime on health and wellbeing, the role of aesthetics and safety from traffic seems to be underestimated, especially in the Polish context. New settlements are often built without sufficient care for green and recreational areas, while the process of suburbanization increases car dependency. These processes are recognized as key constraints to outdoor activity [16], and several studies have shown a positive relationship between the availability of local green space, the health of residents and social cohesion [58,59].

Despite some limitations related to the one-item measures, the results presented are consistent with previous research, and may be considered a confirmation of the construct validity of the NEWS-Poland.

We assume that walkability may be linked to the sense of agency related to life in the city, and all of the subscales, with the exception of residential density, correlated moderately with self-efficacy. Since human activity is considered to be an interplay between behavioural, personal and environmental factors, this study has shed important light on how walkability self-efficacy is related to an adequately designed, pedestrian-friendly environment.

According to Bandura’s theory, motivation and performance are determined by how successful people believe they can be, and how they perceive themselves [60]. Internal beliefs about the ability to fulfil one’s daily needs or move efficiently in one’s neighbourhood may have an impact on events that, in turn, affect their activities. The perception of being in control is an important buffer to negative stress, which is rather unavoidable in the urban context. On the other hand, the perception of being out of control may lead to experiences of distress, even if objective conditions allow the pursuit of goals, and therefore, may elicit maladaptive strategies for responding to stressful situations. As a consequence, people living in less walkable areas who are more prone to experience urban nuisances may develop less efficient strategies for dealing with them. Walkability self-efficacy might play a key role in urban daily functioning, because it affects behaviour not only directly, but also via its impact on other determinants, such as aspirations, goal pursuits and the perception of the environment [61].

In our study, the distance to facilities, street connectivity, aesthetics and safety from crime were all related to local identity. As has been suggested in previous research, certain aspects of walkability may facilitate social interactions, and the development of a more communal identity [62,63]. Public spaces, such as parks and piazzas, may also foster a sense of community by facilitating chance encounters between neighbours [21]; when individuals have a chance to meet each other, they learn social trust [64]. In light of our study, the places that provide easy access to different amenities (close distance to facilities), with connected streets, aesthetics and safety, are more likely to create human-place relationships that stimulate local identity processes. The routine activities of daily life, including repetitive social interactions, are related to places that shape opportunities for experiences of control and predictability. Local identity processes are derived from the relationships with these places, and in a more walkable neighbourhood it is likely easier to incorporate some of the aspects of place to the sense of one’s identity.

In the present study, we compared the districts of Gdansk in Poland, including the historic district and those located on the Lower and Upper Terraces, and we obtained a coherent pattern of results. The historical districts of Gdansk were assessed higher with regard to aesthetics because of their historical buildings (H1—Old Town) and surrounding greenery (H2—Oliwa). Higher walkability has been found among the districts of the Lower Terrace, comparing to the districts of the Upper Terrace. The Lower Terrace was built during the socialist era, according to the modernist standards of a self-sufficient residential urban unit with its own facilities. In contrast, in the Upper Terrace, infrastructure such as services and public spaces is not usually sufficiently provided in this area. Similar to many examples across the world, the districts of the Upper Terrace, which are the subject of intense suburbanization processes, create car dependency, and little ability to meet the daily needs of the neighbourhood. The Upper Terrace has also been rated as less friendly for cyclists and pedestrians, with a lower level of street connectivity and with longer distance to facilities. These aspects describe the common nuisances of life in suburban areas, and confirm the validity of the scale. Despite the fact that both the Upper and Lower Terraces are products of the dominant and criticized idea of functional zoning in urban planning, the Lower Terrace districts are more strongly rooted in the city, located closer to the sea and have a better infrastructure. These conclusions are supported by the results obtained, and we also found relationships between the distance to facilities, land use mix access, street connectivity, places for walking and cycling and proximity to the city centre.

## 5. Limitations

Although the NEWS scale turned out to be a reliable and useful measure of walkability, the limitations of the present study should be highlighted. For example, when it comes to the procedure and participants, some limitations include the fact that this study was conducted online and involved young and middle aged participants, mainly woman, who were self-selected rather than randomly selected. Additional studies are needed in the future among older residents that use the Internet less often, and could be underrepresented in the present on-line research. With regard to the correlations between walkability and the measures of psychological function, we included one-item measures in two cases. This should not be a problem, in and of itself, since the single item measures have been proven to be good indicators of the proposed psychological construct, especially when it comes to subjective self-perception [43]. However, future studies aimed at the replication of the present results could use longer scales to explore the generality of these findings.

## 6. Conclusions

In this study, we have provided some initial evidence for the validity of the NEWS measure adapted for Poland. The findings indicated the satisfactory replication of the factor structure of the scale. To the best of our knowledge, there has been no comprehensive instrument developed to estimate the neighbourhood walkability in Poland thus far. A quantitative approach to the problem of neighbourhood walkability has rarely been undertaken previously, and the discussion about satisfying the residents’ basic needs without the use of cars is still not based on reliable empirical studies. However, we hope that the present scale will be a useful tool for assessing the characteristics of the built environment in a Polish context.

## Figures and Tables

**Table 1:** Standardized factor loadings of the NEWS-PL.

Factors and Items	Standardized Factor Loading
**Land use mix—access (LA)**	
LA1. Many shops within walking distance	0.755
LA2. Many places within walking distance	0.769
LA3. Easy to walk to a public transport stop	0.664
**Street connectivity (SC)**	
SC1. Short distance between intersections	0.628
SC2. Many alternative routes	0.772
**Infrastructure and safety for walking and cycling (IS)**	
IS1. Footpaths on most of the streets	0.657
IS2. Footpaths separated from the road/traffic by parked cars	0.360
IS3. Grass/dirt strip that separates the streets from the footpaths	0.396
IS4. Streets are well lit at night	0.546
IS5. Walkers and bikers easily seen	0.412
IS6. Crosswalks and pedestrian signals	0.543
**Aesthetic (AE)**	
AE1. Trees	0.327
AE2. Many interesting things to look at	0.879
AE3. Attractive natural sights	0.663
AE4. Attractive buildings/homes	0.571
**Traffic safety/hazards (TH)**	
TH1. Heavy traffic along nearby street	0.508
TH2. Slow traffic speed on nearby streets	0.668
TH3. Speeding drivers	0.643
**Safety from crime (CR)**	
CR1. High crime rate	0.898
CR2. Unsafe to walk during the day	0.874
CR3. Unsafe to walk at night	0.956
**Few cul-de-sacs (CS)**	SI
**Physical barriers to walking (BW)**	SI

Note: SI = single item.

**Table 2:** Correlation matrix between NEWS-PL subscales, pleasant affect and local identity (N = 196).

NEWS-PL Subscale	Pleasant Affect		Local Identity	
	*r*	*p*	*r*	*p*
Residential density	−0.08	n.s	0.02	n.s
Distance to facilities	0.03	n.s	0.15	0.031
Land use mix access	0.07	n.s	0.01	n.s
Street connectivity	0.11	n.s	0.16	0.026
Places for walking/cycling	0.10	n.s	0.05	n.s
Aesthetics	0.31	<0.001	0.26	<0.001
Safety from traffic	0.20	0.005	0.00	n.s
Safety from crime	0.35	<0.001	0.16	0.022

n.s: not significant.

**Table 3:** Correlation matrix between NEWS-PL subscales, quality of life, self-efficacy, proximity to the city centre (N = 156).

NEWS-PL Subscale	Quality of Life		Walkability Self-Efficacy		Distance to the City Centre	
	*r*	*p*	*r*	*p*	*r*	*p*
Residential density	−0.04	n.s	0.14	n.s	0.00	n.s
Distance to facilities	0.06	n.s	0.41	<0.001	0.31	<0.001
Land use mix access	0.15	0.056	0.48	<0.001	0.34	<0.001
Street connectivity	0.20	0.012	0.36	<0.001	0.23	0.004
Places for walking/cycling	0.19	0.013	0.39	<0.001	0.24	0.002
Aesthetics	0.28	<0.001	0.41	<0.001	0.13	n.s
Safety from traffic	0.25	0.000	0.36	<0.001	−0.06	n.s
Safety from crime	0.28	<0.001	0.34	<0.001	−0.05	n.s

n.s: not significant.

**Table 4:** NEWS-PL subscale scores among various type of neighbourhood in Gdańsk.

NEWS-PL Subscale	Przymo-rze [LT1]	Zaspa [LT2]	Ujeścisko-Chełm [UT1]	Jasień, Kokoszki, Matarnia [UT2]	Śródmieście [H1]	Oliwa [H2]	*F*	*p*
N	75	64	59	36	21	90		
Residential density	452,73 (108,62)	424,23 (111,06)	295,38 (89,73)	261,94 (57,38)	351.76 (63.75)	353.57 (104.11)	30.85	<0.001
Distance to facilities	3.73 (0.62)	3.45 (0.52)	3.02 (0.58)	2.85 (0.74)	3.42 (0.64)	3.27 (0.56)	15.10	<0.001
Land use mix access	3.58 (0.79)	3.61 (0.59)	3.37 (0.61)	3.19 (0.63)	3.65 (0.73)	3.35 (0.75)	3.03	0.011
Street connectivity	3.11 (0.61)	3.24 (0.57)	3.01 (0.63)	2.61 (0.69)	3.21 (0.56)	3.00 (0.60)	5.47	<0.001
Places for walking/cycling	3.12 (0.43)	3.15 (0.44)	3.05 (0.46)	2.59 (0.64)	2.99 (0.48)	2.96 (0.41)	8.11	<0.001
Aesthetics	2.73 (0.63)	2.72 (0.56)	2.31 (0.54)	2.63 (0.69)	3.10 (0.56)	3.05 (0.60)	13.50	<0.001
Safety from traffic	2.50 (0.54)	2.65 (0.58)	2.91 (0.55)	2.47 (0.72)	2.61 (0.50)	2.77 (0.56)	4.77	<0.001
Safety from crime	2.50 (0.83)	2.77 (0.92)	2.71 (1.09)	2.95 (0.95)	2.52 (0.82)	2.47 (0.94)	2.03	n.s

n.s: not significant.
